# The Use of Trichostatin A during Pluripotent Stem Cell Generation Does Not Affect MHC Expression Level

**DOI:** 10.1155/2022/9346767

**Published:** 2022-02-15

**Authors:** Sara Farahi, Sara Hosseini, Hossein Ghanbarian, Seyed Mahmoud Hashemi, Mohammad Salehi, Samaneh Hosseini

**Affiliations:** ^1^Department of Medical Biotechnology, School of Advanced Technologies in Medicine, Shahid Beheshti University of Medical Sciences, Tehran, Iran; ^2^MOM Fertility & Infertility Research and Innovation Center, Tehran, Iran; ^3^Department of Immunology, School of Medicine, Shahid Beheshti University of Medical Sciences, Tehran, Iran; ^4^Cellular and Molecular Biology Research Center, Shahid Beheshti University of Medical Sciences, Tehran, Iran; ^5^Department of Stem Cell and Developmental Biology, Cell Science Research Center, Royan Institute for Stem Cell Biology and Technology, Tehran, Iran

## Abstract

Pluripotent stem cells (PSCs) are considered as a potent tool for use in regenerative medicine. Highly efficient generation of PSCs through chromatin modulators such as trichostatin A (TSA) might change their MHC molecule expression profile. The efficiency of PSC generation and their immunogenicity is major obstacles for clinical use. Hence, we aim to investigate whether the use of TSA during PSC generation affects MHC expression level. Three PSC lines were generated by iPSCs, NT-ESCs, and IVF-ESCs' reprogramming methods from B6D2F1 mouse embryonic fibroblast cells. Established PSC lines were characterized by alkaline phosphatase assay (ALP) and immunocytochemistry. Their chromosome fidelity was checked by karyotyping. The expression level of pluripotent genes (*oct4, nanog, sox2, klf4*), HDACs (*hdac1, hdac2, and hdac3*), and immune-related genes (including *Qa-1*, *Qa-2*, *H2kb*, *H2kd*, *H2db*, *H2db*, *CIITA*, *H2-IE-βb*, *H2-IE-βd*) in iPSC and ESC lines were assessed by real-time PCR analysis. The presence of MHC molecules on the surface of pluripotent stem cells was also checked by flow cytometry technique. Significant increase of pluripotency markers, *oct4*, *nanog*, *sox2*, and *klf4*, was observed in 100 nM TSA-treated samples. 100 nM TSA induced significant upregulation of *H2db* in generated iPSCs. *H2-IE-βd* was remarkably downregulated in 50 and 100 nM TSA-treated iPSC lines. The expression level of other immune-related genes was not greatly affected by TSA in iPSC and NT-ESC lines. It is concluded that the use of short-term and low concentration of TSA during reprogramming in PSC generation procedure significantly increases PSC generation efficiency, but do not affect the MHC expression in established cell lines, which is in the benefit of cell transplantation in regenerative medicine.

## 1. Introduction

Reprogramming of somatic cells into pluripotent stem cells (PSCs) generates an unlimited number of cells that are able to differentiate into almost all types of cells. PSCs, a valuable cells source in regenerative medicine, are generated by in vitro fertilization (IVF), somatic cell nuclear transfer (SCNT), and reprogramming of somatic cells (i.e., induced PSCs (iPSCs)) [1]. However, their low generation efficiency and potential immunogenicity limit their clinical applications. Thus, it is necessary to improve the efficiency of cellular reprogramming without significant alteration in PSC immune-related gene expression profile.

The establishment of embryonic stem cells (ESCs) from IVF embryos (IVF-ESCs) is affordable and fast, but the obtained ESCs are not genetically identical to the recipient, increasing the risk of graft rejection during cell transplantation. ESCs derived from SCNT established from the cloned embryo provide histocompatible cells for use in regenerative medicine. However, the alloantigenicity of nuclear-transfer ESCs caused by mismatched mitochondria may stimulate host immune response [2, 3]. Autologous iPSCs were under consideration as an ideal candidate to circumvent graft rejection in cell-based therapy up until Zhao et al. published an unexpected report on the immunogenicity of iPSCs derived from syngeneic mice [4]. In addition to the intrinsic immunogenicity of PSCs, the approaches used to improve the efficiency of generation can affect their immunogenicity, which must be considered for further clinical use.

Thus far, a variety of small molecules which target different signaling pathways linked to cell state, function, and epigenetics have been used to elevate the competence of reprogramming [5]. The use of small molecules such as chromatin modulators during the generation of ESC and iPSCs efficiently enhances the expression level of pluripotency genes which in turn improves the rate of reprogramming. DNA methyltransferase inhibitors, histone methyltransferase inhibitors, and histone deacetylase inhibitors (HDI) are currently used in PSC generation to facilitate the reprogramming procedure. Among these modulators, histone deacetylase inhibitors are the earliest factors that have been widely used to increase histone acetylation levels, induce chromatin remodeling, and change the gene expression pattern [6, 7]. Trichostatin A (TSA), a well-known HDI, accelerates the reprogramming procedure by promoting histone acetylation and also decreasing repressive histone methylation in *Sox2*, *C-myc*, and *Oct4* promoters, which are hypermethylated and hypoacetylated in differentiated cells [8, 9]. Since these small molecules cause widespread epigenetic alterations, it may raise concerns about the alteration of immune-related genes, which has been little studied.

Upregulation of immune-related genes such as major histocompatibility complex (MHC) molecules through the reprogramming by chromatin modulators can trigger an immune response during cell transplantation [10]. It has been shown that MHC class II molecules are expressed on the cell surface of a plasma cell tumor following epigenetic activation by the pharmacological agent TSA that makes them act as an efficient antigen-presenting cell to be recognized by immune cells [11]. However, TSA does not impact the MHC expression in a similar way among various cell types because of the difference of three classes of CIITA, the master control factor for the MHC expression, promoters in different cells. Therefore, it is necessary to develop standard and high efficient reprogramming method for having minimal immune-related gene alteration during the generation of clinical-grade embryonic and induced pluripotent stem cells.

In this study, nine ESC lines were generated by IVF and SCNT techniques from B6D2F1 MEF cells. Nine iPSC lines were also originated by Yamanaka's factors from the MEF cells. The efficiency of reprogramming was studied among test groups that were reprogrammed in the presence of different concentrations of TSA and control groups in the absence of TSA. Established cell lines were characterized in terms of morphology, ALP activity, and protein analysis. The genomic integrity of chromosomes was monitored by karyotyping for all cell lines. The expression of pluripotency genes, HDACs, and MHC molecules in different established cell lines was assessed by real-time PCR and flow cytometry.

## 2. Material and Methods

### 2.1. Somatic Cell Nuclear Transfer

Recipient oocytes obtained from B6D2F1 female mice were superovulated by 5 IU of pregnant mare's serum gonadotropin (PMSG) and 5 IU of human chorionic gonadotrophin (hCG) with 48 h intervals [12].14 hours after the last injection, mice were sacrificed, and cumulus-oocyte complexes were incubated in FHM medium with 300 U/ml hyaluronidase. Denuded oocytes were collected and washed in FHM. Enucleation of denuded MII oocytes was performed in KSOM with 5 *μ*g/ml cytochalasin B. Following the enucleation process, oocytes were incubated in KSOM at 37°C. B6D2F1 mouse embryonic fibroblast (MEF) cells were detached by trypsin and washed by PBS three times. Next, they were centrifuged and dissolved in DMEM media. Prepared MEF cells were injected into enucleated oocytes. Following NT, half the oocytes were treated by 100 nM TSA as an experimental group, and the second half remained untreated as a control group. Both groups were placed in a humidified incubator at 37°C in 5% CO_2_ for 2 h to preactivate. In the following procedure, oocytes were activated in 10 mM SrCl2 and 5 g/ml cytochalasin B-supplemented calcium-free KSOM with/without TSA for 6 h [12, 13]. Embryos were remained in KSOM to reach the blastocyst stage (96 h). The numbers of 2-cell, 4-cell, morula embryos, and blastocysts were recorded during 4 days.

### 2.2. Pluripotent Stem Cell (PSC) Generation and Culture

We generated nine iPSC lines (S1, S2, S3, T1, T2, and T3 as TSA^+^ groups, E1, E2, and E3 as TSA^−^ groups) from B6D2F1 MEF cells. In brief, pMXs-Oct3/4, pMXs-c-Myc, pMXs-klf4, and pMXs-sox2 (Addgene) vectors were transfected into Platinum-E (Plat-E) cells by Lipofectamin 3000 (Thermofisher) according to the manufacturer's instructions [14]. Subsequently, viral supernatant was collected, and polybrene added to increase the transduction efficiency. At this time, various concentrations of TSA (50 and 100 nM) were added to the TSA-positive groups. The colonies of iPSCs were picked up on day 10 and expanded. iPSCs were cultured on inactive MEF as a feeder layer. Fresh medium contained DMEM F12 supplemented with 15% KSR, L glutamine (2 mM), sodium pyruvate (1 mM), mLIF (1000 U/ml), 2-mercaptoethanol (100 *μ*M), nonessential amino acid (0.1 mM), and penicillin/streptomycin (50 U). The medium was refreshed every day, and cells were replated twice weekly [15].

Six NT-ESCs and three IVF-ESC lines were also derived from B6D2F1 blastocyst inner cell mass (ICM): S1, S2, and S3 as TSA^+^ groups, F1, F2, and F3 as TSA^−^ groups, and M1, M2, and M3 as control groups. The zona pellucida (ZP) of blastocysts was removed by tyrodic acid (pH = 2.5). Next, ZP-free blastocysts were placed on inactive MEFs. PSCs were initially cultured in an ESC medium as described above. These PSCs were then adapted to feeder-free N2B27 medium.

### 2.3. iPSC and ESC Characterization

#### 2.3.1. Alkaline Phosphatase (ALP) Staining

Alkaline phosphatase detection kit (Sigma-Aldrich 86R-1KT) was used to verify iPSCs colonies. The colonies were fixed by paraformaldehyde 4% for 30 seconds and stained according to the instruction. The images of stained colonies were obtained by Olympus CKX41 microscope with an Olympus DP72 camera.

#### 2.3.2. Immunocytochemistry

The cells were fixed by fresh paraformaldehyde 4% solution at room temperature for 20 minutes. They were washed and permeabilized with 0.05% PBS-tween and 0.7% Triton X-100 and blocked with 2% bovine serum albumin (BSA, Sigma-Aldrich, USA). Cells were incubated with monoclonal mouse IgM anti-human/mouse SSEA1 (cat # MAB2155, R&D System, USA) and mouse oct4 antibody (cat # MAB1759, R&D System, USA) overnight at 4°C. Subsequently, oct4 and SSEA1 were labeled by secondary antibody, Alexa (A11020, Invitrogen, USA) and FITC (cat #71-1900, Invitrogen, USA), respectively, at room temperature for an hour. The nuclei were stained with 4′,6-diamidino-2-phenylindole (DAPI) (D8417, Sigma-Aldrich, USA) [16, 17] and observed by fluorescent microscope (Olympus U-RFL-T, Germany).

#### 2.3.3. Chromosomal Counting

PSCs were treated by colcemid (120 *μ*L diluted in 5 ml culture media) for 90 minutes. Colonies were trypsinized, collected, and exposed to a hypotonic solution that contained sodium citrate and potassium chloride for 20 minutes. The cells were fixed by cold ethanol and gently dropped on a clean glass slide and were stained by Giemsa. The images were captured by Zeiss light microscope, score A1, and analyzed by IKAROS software.

### 2.4. H3lys9 Acetylation

Histone H3 acetyl Lys9 ELISA kit (Active motif, Cat# 53114) was applied to monitor HDAC activity and the amount of histone acetylation in pluripotent stem cells. Histones were extracted by lysis buffer, and the acetylation assay was performed by following the manufacturer's protocol. The absorbance was measured using Thermo Scientific™ Multiskan™ GO Microplate spectrophotometer (Thermo Scientific™, USA) at 450 nm.

### 2.5. RT-PCR and Gene Expression Analysis

Total RNA was extracted by GeneAll kit (314-106, GeneAll Biotechnology Co, Korea) according to the manufacturer's instruction. Complementary DNA was synthesized using Yekta Tajhiz cDNA synthesized kit in two steps. RT-PCR was done for pluripotency genes (*nanog, oct4, klf4*, and *sox2*), MHC genes (*Qa-1*, *Qa-2*, *H2kb*, *H2kd*, *H2db*, *H2dd*, *CIITA*, *H2-IE-βb*, and *H2-IE-βd*), and hdac genes (*hdac1*, 2, and *3*) using Yekta Tajhiz PCR master mix and *β*-actin primer to verify cDNA production. Real-time PCR reactions were performed (StepOne Real-Time PCR system, Applied Biosystems) using a SYBR Green PCR Master Mix (Applied Yekta tajhiz). The relative mRNA expression levels of genes were normalized to those of *β*-actin. mRNA expression levels were calculated using the *ΔΔ*Ct method. Table 1S (Supplementary data) lists all primers used in this study.

### 2.6. Flow Cytometry

The MHC profiling (MHC class I and MHC class II) of pluripotent stem cells and MEF cells was analyzed by flow cytometry. Major histocompatibility complexes were detected using antibodies against MHC class I molecules (MHCI mAb unconjugated, cat # NB120-6405, Novus) and MHC class II molecules (MHCII mAb unconjugated cat # sc-32247, Santa Cruz). Secondary antibody included goat anti-mouse IgG (H + L) antibody (FITC) (cat # orb688924). As negative controls, the cells were stained with appropriate isotype-matched controls. A BD FACS Canto II cell analyzer was used to carry out flow cytometry analysis, and data were analyzed using FlowJo 6.4.7 software.

### 2.7. Statistical Analysis

We compared TSA-treated, nontreated pluripotent stem cell groups and MEFs as the somatic cells using one-way analysis of variance (ANOVA). Analyses were performed with GraphPad Prism 5.0 (GraphPad Software, Inc., San Diego, CA). Data are presented as the mean ± standard error of the mean. *P* value of <0.05 was considered to be significant.

## 3. Results

### 3.1. Generation and Characterization of iPSCs, NT-ESCs, and IVF-ESCs

**Figure 1:**
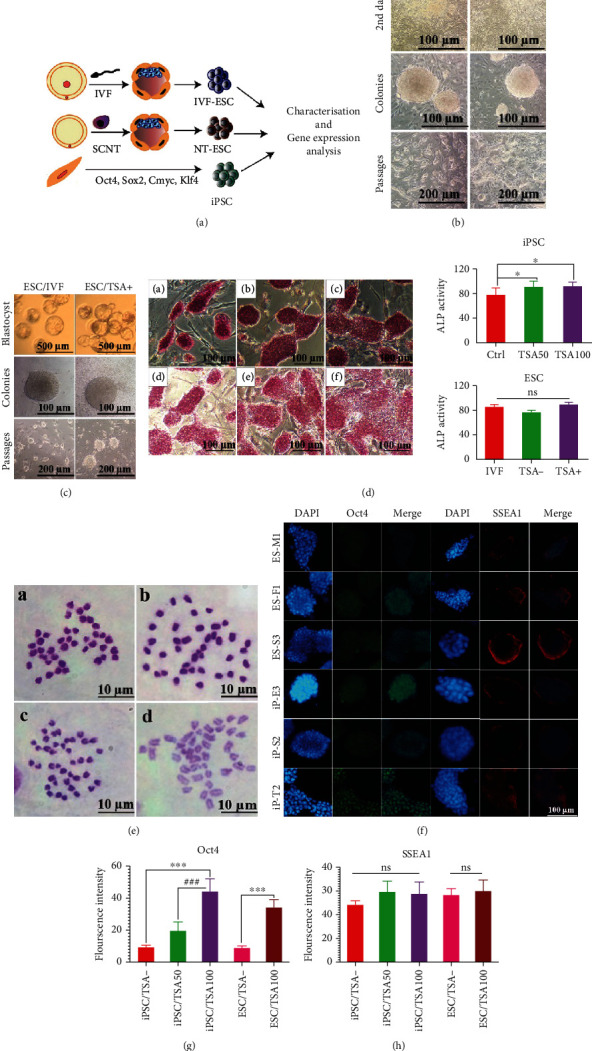
PSCs Characterization. (a) Schematic representation of study design. (b c) All emerged colonies (TSA-iPSCs, TSA+ iPSCs, IVF-ESCs, and TSA+ NT-ESCs) represented typical ESC-like morphology. (d) ALP activity in 100 nM TSA-treated iPSCs (a), 50 nM TSA-treated iPScs (b), TSA-negative iPSCs (c), and ImageJ analysis showed 50 nM and 100 nM TSA concentration significantly increases the ALP expression in generated iPSCs (g). TSA treated ESCs (d), nontreated ESCs (e), and IVF-ESCs (f) all showed high ALP activity (h). (e) Normal karyotype of cell lines was confirmed by karyotyping. (f) The established PSCs express typical pluripotency markers, including OCT4 (green) and SSEA-1 (red). Nuclei were stained with DAPI (blue). (g, h) Fluorescent intensity of OCT4 and SSEA1 in iPSC and ESC colonies was quantified by ImageJ. ^∗^ indicates the statistical significance (*P* < 0.05) compared to the control group. ^∗∗∗^ represents *P* < 0.001.# indicates the statistical significance (*P* < 0.05) between groups.

Schematic representation of the study design is shown in Figure 1(a). Three PSC lines were totally generated by iPSCs, NT-ESCs, and IVF-ESCs' reprogramming methods. During iPSC generation, the morphology of MEFs changed from spindle-shaped to polygonal-shaped cells 24 hours after reprogramming (Figure 1(b), 2^nd^ day). Typical mouse ESC-like colonies appeared 10 days after reprogramming (Figure 1(b), generated colony). Similar rounded-shaped colonies were observed in both TSA-positive groups and control groups. These ESC-like colonies were able to amplify and form new colonies after picking up, and they kept their self-renewal and pluripotency properties after 15 subpassages (Figure 1(b), after 15 passages).

ESC colonies grew out from the inner cell mass of the blastocyst, which was formed by SCNT and IVF procedures (Figure 1(c), blastocyst). Both TSA-subjected and control NT-ESC colonies as well as IVF-ESC colonies showed well-defined boundaries and rounded-shaped morphology (Figure 1(c), generated colony). Like iPSC colonies, they amplified and formed new colonies with self-renewal and pluripotency features after 15 subpassages (Figure 1(c), after 15 passages).

ALP staining was performed for all the cell lines (Figure 1(d)). All the iPSC colonies were turned into red after treatment by ALP's substrate and marinated their activity after passaging (Figures 1(d) and 1(a)–1(c)). Both TSA-treated iPSC groups (50 and 100 nM concentration) showed similar ALP activity patterns yet significantly higher than the control group (*P* value<0.05), as evidence by ImageJ analysis (Figures 1(d) and 1(g)). IVF-ESCs, NT-ESCs, and TSA-treated NT-ESC groups expressed a similar level of ALP (Figures 1(d)–(f) and 1(h)). Of noted, ALP activity was detected in ESC colonies after 15 passages.

Karyotyping analysis indicated that 95% of nontreated established iPSCs preserved their correct chromosome complement of 40 (Figures 1(e) and 1(a)). 80% of TSA-treated iPSCs were remained chromosomally stable throughout 15 culture passages (Figures 1(e) and 1(b)). 90% of IVF-ESCs displayed a normal diploid karyotype set of 40 chromosomes (Figures 1(e) and 1(c)), whereas TSA-negative NT-ESCs and TSA-positive NT-ESCs were appeared to be more variable in chromosome numbers. And they showed 85 and 80% chromosome fidelity, respectively (Figures 1(e) and 1(d) and Table 2S (supplementary data)).

Immunocytochemical staining was performed to evaluate the expression of pluripotent stem cell markers of OCT3/4 and SSEA-1 in the established PSC colonies (Figure 1(f)). ImageJ analysis showed a drastic expression of OCT4 in 100 nM TSA-treated iPSCs followed by 50 nM subjected TSA samples and control groups. Likewise, the expression level of OCT4 dramatically increased in 100 nM TSA-subjected NT-ESCs compared to nontreated NT-ESC colonies (Figure 1(g)).

SSEA1 as a surface marker of mouse pluripotent stem cells was checked among all groups. The same expression level of SSEA1 was detected on the surface of all iPSC and NT-ESC groups (Figure 1(h)).

### 3.2. H3Lys9 Acetylation and HDAC Expression in TSA-Treated Pluripotent Stem Cells

**Figure 2:**
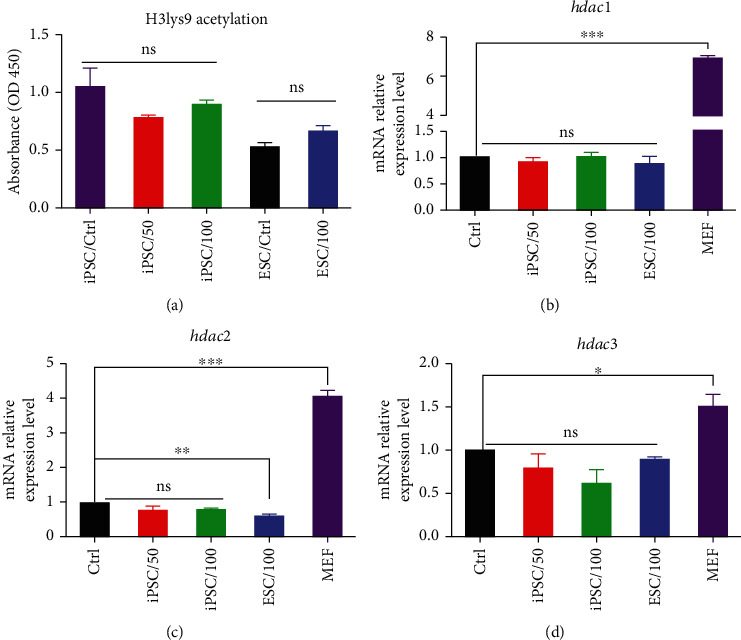
Histone acetylation assay and HDAC expression. (a) Histone acetylation was examined by H3Lys9 acetylation assay for the iPSCs treated with 50 and 100 nM of TSA during reprogramming and 100 nM TSA-treated NT-ESCs. All the groups were compared to the relevant control group. (b)–(d) *hdac* 1, 2, and 3 expressions were assessed by RT-PCR for all test groups and compared to control groups. ns: not significant; ^∗^, ^∗∗^, and ^∗∗∗^ indicate the statistical significance *P* < 0.05, *P* < 0.01, *P* < 0.001, respectively, compared to the control group.

We examined H3lys9 acetylation as a marker of HDAC activity in established ESC and iPSC groups. As can be seen in Figure 2(a), there were no significant differences in H3lys9 acetylation among TSA-positive and TSA-negative groups. We also investigated the *hdacs* gene expression level among established pluripotent stem cells and their parental cells (MEF) in the presence and absence of TSA. RT-PCR results indicated that *hdac* 1, 2, and 3 were expressed at the same level in TSA-treated and nontreated pluripotent stem cells (iPSCs and ESCs). However, MEF cells showed a significant expression of hdac 1, 2, and 3 compared to pluripotent stem cells (*P* < 0.05) (Figures 2(b)–2(d)).

### 3.3. TSA Significantly Improves Reprogramming Rate

**Figure 3:**
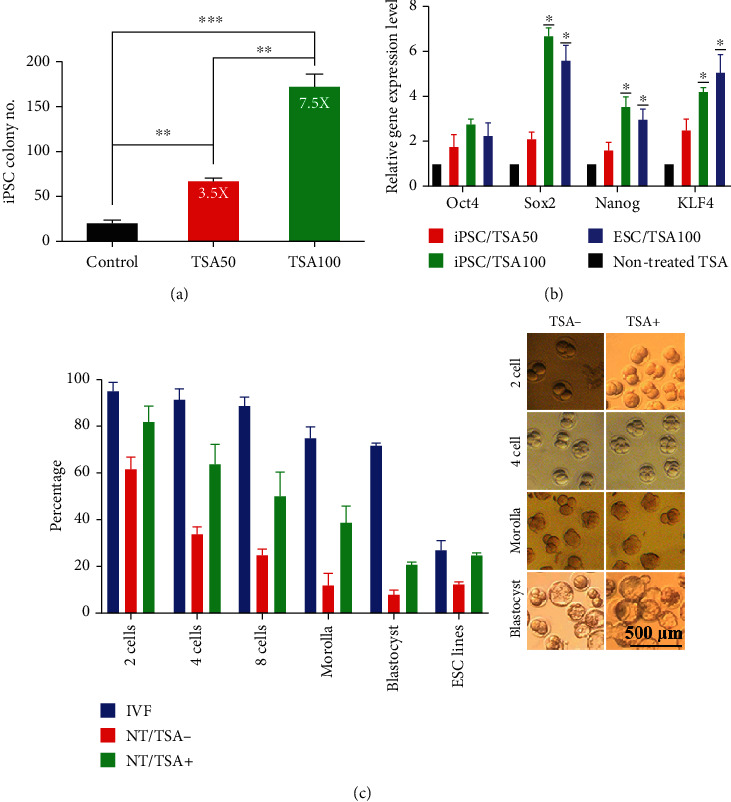
PSC's generation efficiency. (a) iPSC colony numbers showed that TSA treatment increased iPSC colony formation. (b) Pluripotency genes were evaluated by real-time PCR in different samples of iPSCs and ESCs. (c) Rate of embryo development and ESCs generation. ^∗^, ^∗∗^, and ^∗∗∗^ indicate the statistical significance, *P* < 0.05, *P* < 0.01, *P* < 0.001, respectively, compared to the control group.

The total number of appeared iPSC colonies was counted in terms of ALP staining and ESC-like morphology to evaluate each reprogramming method's efficiency. In the iPSC generation procedure, 69 ± 4 ESC-like colonies were counted in nontreated plates at 10 days' posttransduction, while 202 ± 5 colonies appeared in 50 nM TSA-treated plates. Furthermore, colonies number raised up to 518 ± 11 per the same number of seeded cells in 100 nM TSA-subjected plates. 50 and 100 nM of TSA treatment enhanced the efficiency of iPSC generation up to 3- and 7.5–fold, respectively (Figure 3(a)).

Real-time PCR analyses were performed for all cell lines to assess the pluripotency genes (*Oct4*, *Sox2*, *Nanog*, *Klf4*) at the mRNA level. All the pluripotency genes were slightly upregulated in 50 nM TSA-treated iPSC samples compared to control groups, but it was not statistically significant. 100 nM TSA-treated iPSCs displayed a higher expression level of all the pluripotency genes than control groups. However, this increase was just significant for *Sox2*, *Klf4*, and *Nanog*, respectively (*P* < 0.05). Similarly, NT-ESCs showed a drastic rise in *Sox2* and *Klf4* as well as increase in *Nanog*. There was a slight upregulation in *Oct4*, yet not statistically significant (Figure 3(b)).

41 blastocysts were risen up from 57 embryos in IVF procedure (roughly 72%), whereas 97 blastocysts were achieved from 225 embryos by TSA-treated SCNT technique (43%). 28 blastocysts were just grown from 303 embryos following the TSA-free SCNT test. 27.3% of the IVF-blastocyst and 25% of TSA-treated NT-blastocysts turned into ESC colonies when they were placed on the MEF feeder layer, while only 12% of nontreated NT-blastocysts formed ESC colonies(Figure 3(c)).

### 3.4. TSA Treatment during Reprogramming Induces Negligible Changes in MHC Class-I and Class-II Expression

**Figure 4:**
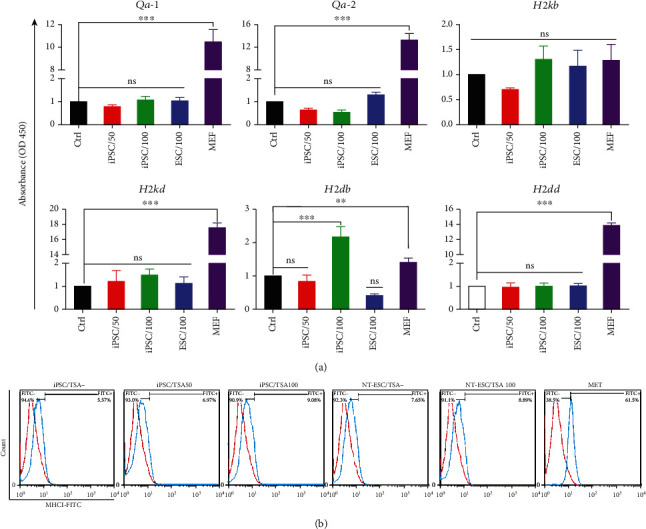
MHC class-I expression level. (a) Histograms shows the real-time PCR analysis of *Qa-1*, *Qa-2*, *H2kb*, *H2kd*, *H2db*, and *H2dd* in TSA-treated ESCs and iPSCs vs. nontreated control groups and parental MEF cells. (b) Flow cytometry analysis of the expression of MHC I molecules on the surface of ESCs, iPSCs, and MEF cell. ns: not significant; ^∗∗^ and ^∗∗∗^ indicate the statistical significance, *P* < 0.01, *P* < 0.001, respectively, compared to the control group.

Real-time PCR analysis showed the expression of *Qa-*1 (MHC class-Ib) slightly decreased in 50 nM TSA-subjected samples that is statistically insignificant. However, it was not altered in 100 nM TSA-treated iPSCs and NT-ESCs. *Qa-*1 expression level was also monitored for the parental somatic cells (MEF) and indicated higher expression level than established pluripotent stem cells (*P* < 0.001) (Figure 4(a)).

TSA treatment during reprogramming caused a decrease of *Qa-*2 (MHC class-Ib) expression in 50 and 100 nM TSA-subjected test groups. The expression level of *Qa-*2 reduced in 50 and 100 nM TSA-treated iPSC groups in a concentration-dependent manner. On the other hand, TSA-subjected NT-ESCs exhibited an increase in the *Qa-*2 gene, which was not statistically significant. MEF cells showed an extreme expression of *Qa-*2 in comparison to ESCs and iPSCs (*P* < 0.001) (Figure 4(a)). We also observed that the SCNT process increased the *Qa-*2 expression level (Figure 1(s), supplementary data).

Similar to *Qa-*1, the expression level of *H2kb* downregulated after the use of 50 nM TSA, while no significant change was detected in 100 nM TSA-treated iPSCs groups and TSA-treated NT-ESCs. Furthermore, MEF cells had the same *H2kb* expression level as ESCs and iPSCs (Figure 4(a)). *H2kb* remarkably decreased in TSA-negative NT-ESCs when compared to IVF-ESCs (Figure 1(s), Supplementary data).

We did not find any significant changes in the expression level of *H2kd* and *H2dd* among all groups. However, MEF cells substantially expressed both *H2kd* and *H2dd* in comparison with ESCs and iPSCs (*P* < 0.0001) (Figure 4(a)). In contrast, the results of the SCNT group indicated significant downregulation of the *H2dd* gene in TSA-negative NT-ESCs (Figure 1(s), Supplementary data).

We did not observe any significant change in the expression level of *H2db* in the 50 nM TSA-treated iPSC group, whereas the use of 100 nM of TSA led to a drastic increase of *HS2db* genes compared to the nontreated group (*P* < 0.001). As expected, *H2db* dramatically expressed in MEF cells (*P* < 0.001) (Figure 4(a)). A significant decline in the expression level of *H2db* occurred in TSA-treated NT-ESC groups (*P* < 0.05). Similar to TSA-treated NT-ESCs, *H2db* dramatically downregulated in nontreated NT-ESC groups (Figure 1(s), Supplementary data).

The MHC expression on ESC and iPSC surface was determined by flow cytometry. 50 and 100 nM TSA-treated groups showed 6.97 and 9.08% MHCI-positive cell population, respectively, while 5.57% of iPS cells in the control group represented MHCI on their surface. 7.65 of TSA negative and 8.89% of TSA-positive ESCs expressed MHC I on their surface. In contrast, 61.5% of MEF cells represented MHC I (Figure 4(b)).

**Figure 5:**
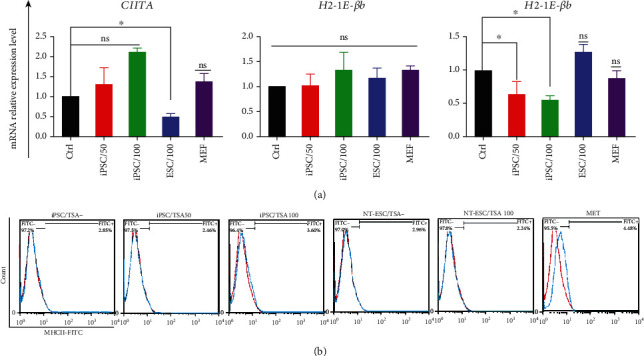
CIITA and MHC class-II expression level. (a) Histograms show the real-time PCR analysis of CIITA, H2-IE*-βb*, and *H2-IE-βd* in TSA-positive and negative iPSCs and ESCs. MEF cells studied as parental somatic cell. (b) Flow cytometry analysis of the expression of MHC II molecules on the surface of ESCs, iPSCs, and MEF cells. ns: not significant; ^∗^ indicates the statistical significance, *P* < 0.05.

Short-term stimulation of reprogramming procedure by TSA slightly increased *CIITA* transcript levels in 50 and 100 nM TSA-treated iPSCs. NT-ESCs showed a dramatic decrease of the *CIITA* expression level compared to the control group. We did not observe any significant expression of CIITA in MEF cells compared to pluripotent cells (Figure 5(a)). *CIITA* expression level was also significantly downregulated among nontreated NT-ESCs (Figure 1(s), Supplementary data).

MHCII genes, including *H2-IE-βb* and *H2-IE-βd*, were evaluated by real-time PCR analysis. As can be seen from Figure 5, no significant changes were observed among the groups in terms of *H2-IE-βb* expression level. On the other hand, *H2-IE-βd* was significantly downregulated in 50 nM and 100 nM TSA-treated iPSCs (*P* < 0.05), while no changes were observed in NT-ESCs and MEF cells compared to control groups (Figure 5(a)).

Flow cytometry assay was also carried out to study the MHC II expression on ESC, iPSCs, and MEF cells' surface. The results showed that 2.46% and 3.6% population of 50 and 100 nM-treated iPSCs expressed MHC II on their surface, respectively, which is similar to TSA-negative iPSCs (2.85%). 2.96% of TSA-negative ESCs and 2.24% of TSA-treated ESCs indicated MHC II on their surface. Only 4.48% of MEF cells were positive for MHC II (Figure 5(b)).

## 4. Discussion

The use of chemical components is one of the strategies for improvement of the PSC generation efficiency. Although many chemical compounds have been suggested to facilitate reprogramming, it is still unclear whether the gene expression profile of PSCs generated by these agents is similar to conventional PSCs [18, 19]. Herein, we used TSA during the generation of iPSCs and NT-ESCs to evaluate the effect of a HDI compound on reprogramming efficiency and MHC expression level.

All the established PSC lines were passaged more than 20 times without differentiation, and they kept their self-renewal features. iPSCs and NT-ESCs represented high ALP activity, round-shape colonies with ESCs-like morphology, and normal karyotype. Immunocytochemistry confirmed upregulation of OCT4 in TSA-treated iPSC samples in a concentration-dependent manner. Although low concentration of TSA is able to upregulate the OCT4 transcription factor as a key point in iPSCs generation, its higher concentration has a profound impact on the OCT4 transcription factor. Upregulation of OCT4 following TSA treatment during reprogramming is apparently associated with stable hyperacetylation of oct4 promoter. Like iPSCs, OCT4 was dramatically upregulated in TSA-treated NT-ESCs, indicating stable acetylation of oct4 promoter even in short-term TSA treatment during SCNT procedure. SSEA1 as a pluripotency surface marker was upregulated in iPSC and NT-ESC lines. And the expression level of SSEA1 was similar in all cell lines, which suggests that all cell lines were successfully reprogrammed to the pluripotent state [20].

TSA facilitates removal of epigenetic memory through reversible inhibition of HDAC enzymes during pluripotent stem cell generation. In this research, the acetylation level of H3Lys9 was not significantly different among TSA-positive and TSA-negative groups which confirmed temporary and reversible effects of TSA aids during the reprogramming procedure. TSA reversibly attaches to the zinc ions in the catalytic site of HDACs; so, after TSA removal, the HDAC catalytic sites and zinc ions become free, and the enzyme activity is restored [21]. Analysis of hdac mRNA level in TSA-treated iPSCs and ESCs revealed that TSA treatment during reprogramming does not affect the hdac expression in derived iPSCs or ESCs, which could prevent the consequentional effect on epigenetics of established iPSCs and ESCs.

According to the colony count results, iPSC colony formation increased by 3- and 7-fold in the presence of 50 and 100 nM TSA, respectively. Blastocyst formation also increased up to 13% in TSA-treated NT-ESC groups. Therefore, TSA as a histone deacetylase inhibitor increased reprogramming efficiency, which is in agreement with the literature [22].

TSA's real-time and steady-state action on the direct histone acetylation and indirect DNA demethylation has been previously confirmed [23]. In our study, real-time PCR analysis showed that pluripotent genes such as *Oct4*, *Sox2*, *Klf4*, and *Nanog* were upregulated in TSA-subjected iPSC samples compared to controls, caused by the stable acetylation of their promoters. Upregulation of pluripotency genes in the TSA-treated iPSCs was concentration-dependent.

Although TSA treatment remarkably enhanced the efficiency of blastocyst formation and ESC origination in the SCNT method, it is still low in comparison with the IVF technique. Similar to iPSC groups, TSA-treated NT-ESC samples represented higher pluripotent genes expression compared to nontreated NT-ESCs, which confirms stable histone acetylation of pluripotent genes' promoters in these cells. These promotors in turn are targeted by transcription factors to enhance gene expression and to help conserve the pluripotency and self-renewal feature in PSCs.

To address the effect of TSA on immune-related genes, we studied the expression level of MHC class I, II, nonclassical MHC, and CIITA in the presence and absence of TSA during iPSC and NT-ESC generation. Transcription of the MHC-I and MHC-II genes in PSCs is mediated by a set of conserved regulatory elements in their promoters, which may be affected by small molecules used to develop reprogramming procedures [24]. Our results revealed that the *Qa-*1 (MHC class-Ib) expression remained unchanged among TSA-treated iPSCs and NT-ESCs compared to their related control groups. *Qa-*1 is a major ligand for the inhibitory receptor CD94/NKG2A and is involved in suppressing NK cells and CD4+ T cells [24] and inducing inhibitory signals to protect *Qa-*1-expressing cells against NK cells. Therefore, it plays an essential role in immune tolerance. The *Qa-*1 expression on PSCs after TSA treatment may help them not to be recognized by the immune system, which is the advantage of this method. RT-PCR analysis determined the downregulation of*Qa-*2 in TSA-treated iPSCs in a concentration-dependent manner which is not significant. However, NT-ESC samples showed a slight increase of *Qa-*2 caused by the SCNT procedure effect not TSA (Figure 1(s), Supplementary data). *Qa-*2 is another member of the MHC Ib family, which can suppress NK cells through interacting Lys49c receptor on NK cells [25, 26] and interfere in immune tolerance. Literature showed that Qa-2 has inhibitory effect even at low expression level and is able to protect the cells against graft rejection [27]. In this study, we found that TSA did not significantly affect the Qa-2 expression which is in the benefit of cell therapy. However, more research on the MHC Ib expression on these cells and their differentiated derived cells is needed to clearly address the mechanism of protecting ESC and iPSCs from NK cell.

Short-term treatment of TSA in the iPSC generation process did not affect *H2kb*, *H2kd*, and *H2dd* expression, which might relate to unstable acetylation of their promoters in these cell lines. Of noted, stem cell researches have shown that the expression of MHC class I and nonclassical MHC-I molecules considerably decreases on PSC's surface, which protects them from T and B cell recognition. Although it would be beneficial in cell-based therapy, the absence of MHCI and nonclassical MHC on PSC's surface would susceptible these cells to be rejected by natural killer cells [10, 28]. A minimum expression of MHC molecules on the PSCs' surface is thus necessary to fail their rejection by the immune system. Additionally, numerous cancer research groups have investigated the impact of TSA on MHC molecules, as HDI agents are used in chemotherapy. These studies revealed that TSA could potentially increase tumor immunogenicity through the overexpression of MHC I molecules on the cancer cells, and it raises the concern about using TSA in reprogramming procedures [10]. TSA inhibits histone deacetylase class I (HDACI) enzyme activity, which induces the MHC I expression. The permanent use of TSA in PSC culture media also enhanced the expression of MHCI molecules on the PSC surface.

Contrary to previous studies, our results revealed that the mRNA expression level of *H2kb*, *H2kd*, and *H2dd* remained unchanged among different cell lines generated in the presence of 50 and 100 nM concentrations of TSA [29, 30]. It is believed that the use of TSA for a short period of time during the reprogramming procedure does not induce stable acetylation of *H2kb*, *H2kd*, and *H2dd* promoters. Surprisingly, we observed the drastic increase of *H2db* expression level by the addition of 100 nM TSA in iPSC lines, which may probably be related to the increase of *H2db's* promoter acetylation caused by TSA. TSA has different effects on the gene expression and selectively induces acetylation of different promoters, which inhibit/stimulate different gene expressions in permanent/temporary order [23]. Accordingly, various haplotypes may respond differently to TSA. Unlike iPSCs, the expression level of *H2db* reduced in TSA-treated NT-ESCs, which is allocated to SCNT procedure but not TSA effects as *H2db* downregulated in nontreated NT-ESCs compared to IVF-ESCs (supplementary data, Figure 1).

Short-term use of 50 and 100 nM of TSA during reprogramming procedure slightly upregulated *CIITA* expression level in a concentration-dependent manner. Different studies highlighted the importance of *CIITA* in regulating MHC class II expression by recruiting histone-modifying enzymes and transcription factors. *CIITA* is regulated by DNA methylation and histone modifications at the transcription level [10, 31]. The alteration of the *CIITA* expression level may change the expression of downstream MHC II molecules. The use of a high concentration of TSA during iPSC generation upregulates the *CIITA* expression and is not recommended in clinical approaches. Surprisingly, 100 nM TSA-treated NT-ESC samples showed a significant reduction of the *CIITA* expression. The comparison of TSA-negative NT-ESCs, TSA-positive NT-ESCs, and their control IVF-ESCs revealed that a significant decrease of *CIITA* was related to the SCNT procedure (Figure 1(s), Supplementary data). Therefore, TSA administration for a short time during the SCNT procedure would decrease *CIITA* expression level, which subsequently may lead to downregulation of MHC II molecules. The MHCII molecules, including *H2-IE-βb* and *H2-IE-βd*, are restricted to antigen-presenting cells (APC).

In contrast to *CIITA*, *H2-IE-βb* did not undergo any changes in TSA-treated iPSCs, which indicates that the slight increase of *CIITA* at the transcription level might not affect the *H2-IE-βb* expression level. Surprisingly, MHC II genes did not change in NT-ESCs, which may occur either by SCNT procedure that could induce the gene expression (Figure 1(s), Supplementary data), or the direct effect of acetylation caused by TSA on *H2-IE-βb* and *H2-IE-βd* promoters, which resist against *CIITA* downregulation. Unexpectedly, *H2-IE-βd* downregulated in TSA-treated iPSCs in a concentration-dependent manner indicated that TSA might inhibit the *H2-IE-βd* expression. Moreover, flow cytometry results confirmed that all the iPSC and ESC groups expressed MHC I and II at the low level. No significant changes were observed among the test groups which is in line with the RT-PCR results. Therefore, it is believed that TSA aids during reprogramming would not affect MHC presence on generated PSCs at the protein level.

## 5. Conclusion

Highly efficient and accurate establishment of PSC lines is of utmost importance in cell-based therapies. TSA as an HDI significantly increases the reprogramming rate. However, its effects on immune-related gene expression have remained unclear. This study investigated the effect of TSA treatment on MHC molecules and CIITA expression in iPSCs and NT-ESCs. Our results showed that nonclassical MHC was not affected by TSA among TSA-treated iPSC and ESC groups. Among MHC I genes, only the *H2db* haplotype increased in the presence of 100 nM TSA, while other MHCI molecules remained unchanged. The expression level of the CIITA gene increased in TSA-subjected iPSCs in a concentration-dependent manner. TSA did not influence MHC II in all established cell lines. To sum up, TSA treatment during PSC generation increases the rate of reprogramming through pluripotency gene upregulation. Low concentration and temporary use of TSA during reprogramming procedure do not greatly affect MHC molecule expression in PSCs that is in favor of cell replacement therapy.

## Data Availability

The data sets supporting the conclusions of this article are included within the article and its supplementary files.

## Supplementary Materials

Supplementary Materials Figure 1(s) (Supplementary data): ESC immune-related gene expression in presence and absence of TSA compared to IVF-ESCs as control. Qa-1 significantly decreased in TSA-negative NT-ESCs in compare to IVF-ESCs and TSA-positive NT-ESCs. Qa-2 displayed a slight increase in both test groups. H2kb dramatically reduced in nontreated TSA NT-ESCs in compare to both IVF-ESCs and TSA-treated NT-ESCs. H2kd did not show any changes in test groups. Drastic decrease of H2db was observed in TSA-treated and nontreated NT-ESCs. SCNT procedure induced substantial reduction of H2dd in TSA-negative NT-ESCs in compare to IVF and TSA-subjected ESCs. CIITA exhibited dramatic reduced in both TSA-treated and nontreated NT-ESCs. H2-IE-*β*b had the same expression in all cell lines. H2-IE-*β*d significantly increased in TSA-negative NT-ESCs in compare to TSA-positive and IVF ESCs. Table 1S (Supplementary data): primers for real-time polymerase chain reaction analysis (real-time PCR) are listed. Table 2S (Supplementary data): karyotyping analysis. Chromosome number of different cell lines analyzed by karyotyping. TSA-negative iPSCs and ESCs showed 95 and 90% normal karyotype, respectively. 80% of TSA treated iPSC lines preserved their correct chromosome complement of 40. 85% of TSA-negative NT-ESCs displayed a normal diploid karyotype set of 40 chromosomes. And 80% of TSA-positive NT-ESCs showed stable chromosome set.
