# Supplementary material for: The Use of Trichostatin A during Pluripotent Stem Cell Generation Does Not Affect MHC Expression Level
Source: Stem Cells Int. 2022 Feb 15;2022:9346767. doi: 10.1155/2022/9346767 (PMC8967593; doi:10.1155/2022/9346767)
Supplement: Supplementary Materials — Figure 1(s) (Supplementary data): ESC immune-related gene expression in presence and absence of TSA compared to IVF-ESCs as control. Qa-1 significantly decreased in TSA-negative NT-ESCs in compare to IVF-ESCs and TSA-positive NT-ESCs. Qa-2 displayed a slight increase in both test groups. H2kb dramatically reduced in nontreated TSA NT-ESCs in compare to both IVF-ESCs and TSA-treated NT-ESCs. H2kd did not show any changes in test groups. Drastic decrease of H2db was observed in TSA-treated and nontreated NT-ESCs. SCNT procedure induced substantial reduction of H2dd in TSA-negative NT-ESCs in compare to IVF and TSA-subjected ESCs. CIITA exhibited dramatic reduced in both TSA-treated and nontreated NT-ESCs. H2-IE-βb had the same expression in all cell lines. H2-IE-βd significantly increased in TSA-negative NT-ESCs in compare to TSA-positive and IVF ESCs. Table 1S (Supplementary data): primers for real-time polymerase chain reaction analysis (real-time PCR) are listed. Table 2S (Supplementary data): karyotyping analysis. Chromosome number of different cell lines analyzed by karyotyping. TSA-negative iPSCs and ESCs showed 95 and 90% normal karyotype, respectively. 80% of TSA treated iPSC lines preserved their correct chromosome complement of 40. 85% of TSA-negative NT-ESCs displayed a normal diploid karyotype set of 40 chromosomes. And 80% of TSA-positive NT-ESCs showed stable chromosome set. [file 9346767.f1.docx]

**Supplementary Data**

**The Use of Trichostatin A during Pluripotent Stem Cell Generation Does Not Affect MHC Expression Level**

Sara Farahi^1^, Sara Hosseini^1^, Seyed Mahmoud Hashemi^2^, Hossein Ghanbarian^1,3^, Mohammad Salehi^1,3*^, Samaneh Hosseini^4*^

^1^ Department of Medical Biotechnology, School of Advanced Technologies in Medicine, Shahid Beheshti University of Medical Sciences, Tehran, Iran

^2^Department of Immunology, School of Medicine, Shahid Beheshti University of Medical Sciences, Tehran, Iran

^3^Cellular and Molecular Biology Research Center, Shahid Beheshti University of Medical Sciences, Tehran, Iran

^4^ Department of Stem cell and developmental Biology, Cell Science Research Center, Royan Institute for Stem Cell Biology and Technology, Tehran, Iran

^*^Corresponding Authors:

Mohamad Salehi, email: [Msalehi78@gmail.com](mailto:Msalehi78@gmail.com). Tel: +982122439956, Fax: +982189784665

Samaneh Hosseini, email: [Hosseini.samaneh@royainstitute.org](mailto:Hosseini.samaneh@royainstitute.org). Tel: +982123562512, Fax: +982123562507

| Gene name | **Forward prime (5´-3´)** | **Reverse primer (5´-3´)** | **Accession number** |
| --- | --- | --- | --- |
| *Qa-1* | TAT TGG GAG CGG GAG AC | CGT GTG AGA TTC GTC GTT AC | NM_010398.3 |
| *Qa-2* | GGA CAT GGA GCT TGT GGA G | CCT TGT CCA CCC CAT CTC | NM_001198560.1 |
| *H2kb* | CTC CCA GAT TGT AAA GTG ATG | CAC TTG ACT AAA GAG AAC TGA GG | NM_001001892.2 |
| *H2kd* | TCA TCG CTG TCG GCT AC | ACT GCT CAT CGC TCT TGG | NM_001370818.1 |
| *H2dd* | GAG GAC CGC ACA GAG ATA C | GTC GTA GGC GAA CTG CC | NM_023124.5 |
| *H2db* | GAG ATT GTA AAG CGT GAA GAC | TGT TGT GTA AAG AGA ACT GAG G | NM_010380.3 |
| *CIITA* | CCA ACA TTG CGG AAC TG | CCT GTG CTT TGA GTC CAT AG | NM_001302619.1 |
| *H2-IE-βd* | GAT GTT GAG TGG AGT TGG G | AAG TCC AGA CTG TCT TTC TGG |  |
| *H2-IE-βb* | TCC ACC CAC CTG CTT TC | CAC AAG AGT CAA GAG GAA TAC C |  |
| *oct4* | GGC GTT CTC TTT GGA AAG GTG TTC | CAT ACT CGA ACC ACA TCC TTC TCT |  |
| *nanog* | CCT ATT AAG GTG CTT GCT TGT C | TCG GTT CAT CAT GGT ACA GTC | NM_028016 |
| *sox2* | GCT GGG AGA AAG AAG AGG AG | ATC TGG CGG AGA ATA GTT GG | NM_011443.3 |
| *klf4* | AGT TCT CAT CTC AAG GCA CA | CTT CAT GTG TAA GGC AAG GT |  |
| *B-actin* | CTT CTT GGG TAT GGA ATC CTG | GTG TTG GCA TAG AGG TCT TTA C | NM_007393.5 |

**Table 1S** Primers for Real-time polymerase chain reaction analysis (Real-time PCR) are listed.

**
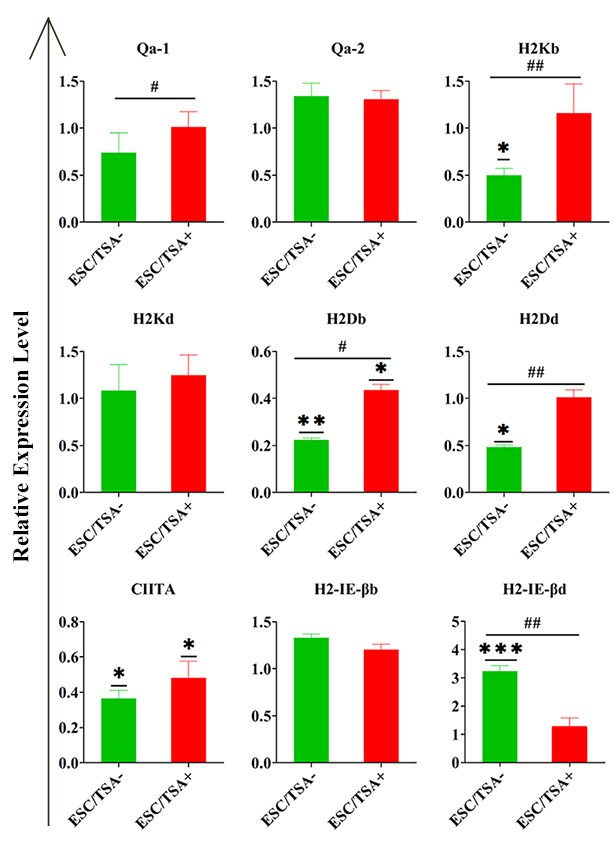
**

**Figure 1S ESCs immune-related gene expression in presence and absence of TSA compared to IVF-ESCs as control.** *Qa-1* significantly decreased in TSA negative NT-ESCs in compare to IVF-ESCs and TSA positive NT-ESCs. *Qa-2* displayed a slight increase in both test groups. *H2kb* dramatically reduced in non-treated TSA NT-ESCs in compare to both IVF-ESCs and TSA-treated NT-ESCs. *H2kd* didn’t show changes in test groups. Drastic decrease of *H2db* was observed in TSA-treated and non-treated NT-ESCs. SCNT procedure induced substantial reduction of *H2dd* in TSA negative NT-ESCs in compare to IVF and TSA-subjected ESCs. CIITA exhibited dramatic reduced in both TSA-treated and non-treated NT-ESCs. *H2-IE-βb* had the same expression in all cell lines*. H2-IE-βd* significantly increased in TSA negative NT-ESCs in compare to TSA positive and IVF ESCs.

|  | ESC lines |  | |  | iPSC lines |  |  |
| --- | --- | --- | --- | --- | --- | --- | --- |
| NT-ESCs/ TSA+ | NT-ESCs/ TSA- | IVF-ESCs | iPSC/TSA100 | | iPSC/TSA50 | iPSC/TSA- | Cell Numbers |
| 28 | 40 | 40 | 31 | | 40 | 40 | 1 |
| 40 | 40 | 40 | 40 | | 38 | 37 | 2 |
| 40 | 40 | 40 | 40 | | 40 | 40 | 3 |
| 40 | 40 | 40 | 39 | | 41 | 40 | 4 |
| 40 | 40 | 40 | 40 | | 40 | 40 | 5 |
| 40 | 40 | 40 | 40 | | 40 | 40 | 6 |
| 40 | 40 | 40 | 40 | | 40 | 40 | 7 |
| 32 | 52 | 25 | 40 | | 40 | 40 | 8 |
| 40 | 40 | 40 | 40 | | 39 | 40 | 9 |
| 40 | 40 | 40 | 40 | | 40 | 40 | 10 |
| 40 | 40 | 40 | 40 | | 40 | 40 | 11 |
| 42 | 40 | 40 | 35 | | 40 | 40 | 12 |
| 40 | 40 | 40 | 40 | | 40 | 40 | 13 |
| 40 | 40 | 40 | 40 | | 40 | 40 | 14 |
| 40 | 40 | 40 | 40 | | 37 | 40 | 15 |
| 39 | 29 | 40 | 40 | | 40 | 40 | 16 |
| 40 | 40 | 40 | 38 | | 40 | 40 | 17 |
| 40 | 40 | 40 | 40 | | 40 | 40 | 18 |
| 40 | 40 | 40 | 40 | | 40 | 40 | 19 |
| 40 | 39 | 40 | 40 | | 40 | 40 | 20 |
| 80% | 85% | 90% | 80% | | 80% | 95% |  |

**Table 2S Karyotyping analysis.** Chromosome number of different cell lines analyzed by karyotyping. TSA negative iPSCs and ESCs showed 95 and 90% normal karyotype, respectively. 80% of TSA treated iPSC lines preserved their correct chromosome complement of 40. 85% of TSA-negative NT-ESCs displayed a normal diploid karyotype set of 40 chromosomes. And 80% of TSA-positive NT-ESCs showed stable chromosome set.
